# The transgenerational study of insulin action in female offspring adult Wistar rats

**DOI:** 10.1186/1758-5996-7-S1-A128

**Published:** 2015-11-11

**Authors:** Eduardo Kloppel, Yuri Karen Sinzato, Debora Cristina Damasceno, Gustavo Tadeu Volpato, Kleber Eduardo de Campos

**Affiliations:** 1 UFMT, Barra do Garças, Brazil

## Background

Obesity is related with the most present patophysiology disturbance in population, mainly in women, being as an important factor to glucose metabolic changes. Among the etiological factors, it is known the transgeracional effect of obesity allow this syndrome be developed in further generations, without genetic interference.

## Objective

To evaluate the insulin secretion and action profile in adult age of rats from a gestational obesity.

## Materials and methods

Twelve newborn female Wistar rats were used, and half of them submitted to saline solution administration (control) and the other half were administrated monosodium glutamate solution, 4.0 mg/Kg body weight (obese) in neonatal period. At adult age (90 days of life) these female rats were mated with health male rats and the female offspring were used, divided into two groups: control (CONT, n=28) and obese (OB, n=15), according to its previous dam group. In all adult age (from 3rd to 7th months) the rats were monthly evaluate the Lee Index, water and food intake, 12h-fasting glycemia, oral glucose tolerance test (OGTT) and insulin test tolerance (ITT). In addition, from OGTT Results it was estimated the area under the glycemic curve (AUC). All data were statistically analyzed with 5% significance.

## Results

The CONT female rats presented as normal by Lee Index only in 3rd month; and obese from 4th to 7th month in CONT group and also all months evaluated in OB group. In CONT rats, water intake was increased in months 6 and 7 and food intake in all months. The OB rats showed increasing in water intake (months 5 to 7) and food intake (months 4, 6, 7), all compared to 3rd one. The glycemic levels was increased from 5th to 7th in CONT group, whereas it has increased at 6th and 7th months in OB rats, and between groups the female rats presented higher glycemia in months 3, 6 and 7 compared to CONT ones. The insulinic tests (OGTT and ITT) in all months of CONT group showed as normal. Therefore, at 7th month in OB group showed glucose intolerance (glucose increasing in timepoints 30′, 60′ and 120′ in OGTT, compared to fasting, with increasing AUC) and insulin resistance (no differences at timepoints 15′, 30′, and 60′ in ITT, compared to fasting).

## Conclusion

The gestational period associated with obesity leads glucose intolerance and insulin resistance associated to aging process, confirming the transgerational effect in female rats with changes of insulin action in peripheral tissues.

